# Identifying the Target Traumatic Brain Injury Population for Hyperbaric Oxygen Therapy

**DOI:** 10.3390/ijms241914612

**Published:** 2023-09-27

**Authors:** Samantha Schimmel, Bassel El Sayed, Gavin Lockard, Jonah Gordon, Isabella Young, Francesco D’Egidio, Jea Young Lee, Thomas Rodriguez, Cesar V. Borlongan

**Affiliations:** 1 Morsani College of Medicine, University of South Florida, 560 Channelside Dr., Tampa, FL 33602, USA; samanthaschimmel@usf.edu (S.S.); belsayed@usf.edu (B.E.S.); gavinlockard@usf.edu (G.L.); jonahgordon@usf.edu (J.G.); 2 University of Arkansas, Fayetteville, AR 72701, USA; iyoung00@icloud.com; 3 Center of Excellence for Aging and Brain Repair, Department of Neurosurgery and Brain Repair, Morsani College of Medicine, University of South Florida, 12901 Bruce B Downs Blvd, Tampa, FL 33612, USA; francescodegidio@usf.edu (F.D.); jeayoung@usf.edu (J.Y.L.); 4 School of Medicine, Loma Linda University, 11175 Campus St., Loma Linda, CA 92350, USA; trodriguez862@gmail.com

**Keywords:** traumatic brain injury, chronic impairments, hyperbaric oxygen therapy

## Abstract

Traumatic brain injury (TBI) results from direct penetrating and indirect non-penetrating forces that alters brain functions, affecting millions of individuals annually. Primary injury following TBI is exacerbated by secondary brain injury; foremost is the deleterious inflammatory response. One therapeutic intervention being increasingly explored for TBI is hyperbaric oxygen therapy (HBOT), which is already approved clinically for treating open wounds. HBOT consists of 100% oxygen administration, usually between 1.5 and 3 atm and has been found to increase brain oxygenation levels after hypoxia in addition to decreasing levels of inflammation, apoptosis, intracranial pressure, and edema, reducing subsequent secondary injury. The following review examines recent preclinical and clinical studies on HBOT in the context of TBI with a focus on contributing mechanisms and clinical potential. Several preclinical studies have identified pathways, such as TLR4/NF-kB, that are affected by HBOT and contribute to its therapeutic effect. Thus far, the mechanisms mediating HBOT treatment have yet to be fully elucidated and are of interest to researchers. Nonetheless, multiple clinical studies presented in this review have examined the safety of HBOT and demonstrated the improved neurological function of TBI patients after HBOT, deeming it a promising avenue for treatment.

## 1. Introduction to TBI

Traumatic brain injury (TBI) may involve direct penetrating and indirect non-penetrating force to the head [1,2,3]. While direct penetrating TBI consists of a direct mechanical blow to the head, indirect non-penetrating TBI occurs when an external force, not necessarily acting directly on the head, is partly absorbed by the brain because of sudden acceleration–deceleration phenomena, such as in blast shock waves, high-speed car collisions, concussions, among others [1,2,3]. In addition to this primary penetrating and non-penetrating brain injury, TBI triggers secondary brain injury comprising a constellation of pathologies, including focal intracranial hemorrhages and cerebral edema, which may exacerbate the primary brain injury [4,5]. Accordingly, when a patient requires neurocritical care after a TBI, both primary and secondary brain injuries should be given utmost consideration [4]. As defined above, the primary brain injury may involve either direct penetrating or indirect non-penetrating forces that occur at the time of the traumatic impact on the brain tissue [1,2,3]. These forces and the injury they cause to the brain tissue initiate a cascade of cell death events, altogether referred to as secondary brain injury, over time. The impact of secondary brain injury caused by dysautoregulation of brain vessels and blood–brain barrier (BBB) disruption may magnify the primary brain injury, culminating in the development of brain edema, elevated intracranial pressure, and finally, reduced cerebral perfusion pressure [5,6]. Both primary and secondary brain injury processes are accompanied by many clinical factors, such as the depolarization and disturbance of ionic homeostasis, neurotransmitter release (e.g., glutamate excitotoxicity), mitochondrial dysfunction, neuronal apoptosis, lipid degradation, and the initiation of inflammatory and immune responses [5,6]. This extremely complex nature of primary and secondary brain injury mechanisms makes it difficult to simply and clearly differentiate between these pathological factors in patients with TBI [7,8]. The clinical presentation of TBI varies greatly, but common symptoms include altered mental status, headache, nausea, vomiting, and even focal deficits such as hemiparesis (paralysis affecting one side of the body), decreased sensation, and cranial nerve dysfunction. Following a primary survey (airway, breathing, circulation), patients should have their severity categorized utilizing the Glasgow Coma Scale (GCS) [6,7], which assesses brain function by examining the eyes, speech, and motor. A mild TBI is a GCS 13–15; moderate TBI 9–12; and a severe TBI ≤ 8, which typically indicates the need for endotracheal intubation.

Though imaging is important in diagnostics, neuroprotective measures should be pursued foremost to avoid secondary brain injuries such as blood brain barrier (BBB) disruption, including actively managing blood oxygen and carbon dioxide, glucose, temperature, and blood pressure. The prognosis of these patients depends on the initial severity. Some 80–90% of patients with mild TBIs experience complete recovery within 2 weeks, though post-concussion syndrome can result in lingering symptoms [8]. Some 90% of patients with moderate TBIs improve, but 44% continue to suffer from moderate disability. Unfortunately, 10% decline to severe TBI [9]. Those with severe TBIs face up to 35% mortality rate, though this rate decreases with early transfer to neurocritical units [10]. Ultimately, TBIs present with a constellation of symptoms deriving from various etiologies, and prognoses range from recovery, chronic symptoms affecting quality of life, and up to death.

## 2. HBOT in the Setting of Brain Injury

Hyperbaric oxygen therapy (HBOT) is defined as the administration of 100% oxygen at a pressure above 1 ATM and is being increasingly utilized in the treatment of several medical conditions. The central mechanism of HBOT revolves around enhanced oxygen delivery to tissues, thereby increasing the production of reactive oxygen species (ROS) and reactive nitrogen species (RNS) and, in turn, leading to diminished inflammatory responses, improved neovascularization, increased wound growth factor synthesis, and enhanced antibacterial effects [11,12]. In the context of TBI, HBOT has a theoretical role in improving the hypoxia causing secondary brain injury; with insufficient oxygenation, metabolic homeostasis is lost within neurons leading to the accumulation of free oxygen radicals and membrane degradation [13]. Research into the applications of HBOT in TBI dates back to the 1960s, when Coe and Hayes reported neuroprotective effects in rats subjected to experimental brain injury [14]. Subsequent studies have since found benefits related to a reduction in cerebral edema, intracranial pressure (ICP), apoptosis, and inflammation while increasing brain tissue pO_2_, cerebral glucose utilization, vascular density, and synaptic remodeling, each of which has effects on improved functional and cognitive outcomes [14,15,16,17,18,19,20,21,22,23,24,25,26,27,28].

Research has also centered on the potential therapeutic time window of HBOT following TBI and suggests a role in both the acute and chronic setting. Within 24 h of injury, HBOT appears to promote neuroprotection through the regulation of mitochondrial permeability and reduction of neuroinflammation [18,19]. Further, multiple sessions administered up to 48 h following injury, rather than a single session, significantly reduce acquired neurological deficits [29]. Other studies have demonstrated that repetitive long-term HBOT improves motor function recovery in rat models, and even when initiated at 50 days following injury enhances hippocampal recovery and overall cognitive function [23,26]. Pre-clinical work has also begun to demonstrate translational applicability to clinical medicine. Two Phase I trials found sustained improvement in intracranial pressure after HBOT in both comatose and non-comatose samples, with improvement in awareness and motor activity in the trial involving comatose patients [30,31]. Phase II trials have yielded less clear results, with some studies reporting improved Glasgow Outcome Scale (GOS) scores, length of hospital stay, behavior, disability rates, and mortality rates [32,33,34,35], while another found no significant difference in GOS scores [36]. However, even without an improvement in GOS scores, this particular study still reported a 50% relative reduction in mortality [36]. The overall evidence for HBOT remains compelling despite previous inconsistent clinical findings, and thus research continues into the potential role of enhancing oxygen delivery for improvement in metabolic function and intracranial hypertension following TBI (Table 1). A comprehensive review of pertinent military-funded clinical trials is detailed in Table 2.

## 3. Chronic Impairments in TBI Populations

When evaluating whether HBOT is indicated for TBI, one must note the heterogeneity underlying such brain injuries. As mentioned above, clinicians and scientists classify TBI as either mild (mTBI), moderate, or severe. mTBI accounts for the vast majority of TBIs, with an estimated 80–90% of brain injuries fitting this classification [62,63]. Typically, mTBI has been viewed as an acute injury, with most neurocognitive and behavioral deficits resolving within days to weeks [64]. This is in contrast to moderate and severe TBIs, which are known to cause progressive morbidity and mortality in the estimated 500 out of 10,000 Americans who suffer from them annually [62]. While the exact deficits vary based on the mechanism of injury and individual characteristics, many patients report neurocognitive dysfunction, prolonged fatigue, and psychiatric disturbances, including but not limited to impulsivity, irritability, and depression [62,65]. Recent evidence indicates that those with mTBI may also experience subtle chronic symptoms, including impaired executive functioning, somatic dysfunction, and personality changes [64,66]. Such changes following mTBI are referred to as persistent post-concussion symptoms (PPCS) and are likely under-reported [66,67]. Furthermore, certain TBI etiologies may impart a greater risk of prolonged symptoms. Veterans are especially susceptible to TBI and PPCS, with an estimated 12–20% of those who fought in Iraq and/or Afghanistan experiencing one or more mTBI [64]. In an autopsy study on U.S. military veterans with a history of blast-related TBI, Goldstein et al. found that those suffering from blast-induced TBI had the morphological characteristics of chronic traumatic encephalopathy (CTE), a progressive brain disorder characterized by neuroinflammation, microvascular injury, neurodegeneration, and concurrent clinical symptoms [68]. Given the clinical and societal burden of PPCS and CTE, identifying those at risk remains an important step in improving patient outcomes.

While the pathophysiology underlying TBI has yet to be fully elucidated, those who suffer from TBI experience both primary and secondary neurological injury. The primary injury refers to the initial TBI causing insult and results in diffuse irreversible damage to neurons, glia, and the cerebral vasculature [69]. Secondary injury refers to the subsequent biochemical and cellular changes and can last for years [70]. The hallmarks of secondary injury include altered neuronal and glial morphology, excitotoxicity, neuroinflammation, demyelination, and oxidative stress, all of which may serve as future targets for improving post-TBI symptoms and functioning [71,72]. Neuroinflammation in particular may represent a targetable process, as the influx of monocytes and other inflammatory cells into areas of damage has been well established. Animal studies have shown that blocking the infiltration of these inflammatory cells results in a marked reduction in both neurodegeneration and cognitive deficits [73,74]. Another pathophysiology of secondary injury following TBI relates to dysregulated cerebral blood flow (CBF) [69]. Given the high metabolic demands of neural tissue, even slightly dysregulated CBF can result in hypoxic changes that trigger cell death cascades [75]. While further research into the exact mechanisms underlying HBOT in TBI is warranted, the aforementioned ability of HBOT to attenuate both neuroinflammation and hypoxia makes it promising in the reduction of chronic neuropathology.

## 4. HBOT Mechanism of Action

Regarding the mechanism of action underlying HBOT, studies have shown reduced levels of edema, inflammation, and apoptosis along with increased levels of angiogenesis and neurogenesis after HBOT [71]. While the mechanisms mediating these effects are not fully understood, several studies have identified potential pathways. Specifically, the therapeutic effects of HBOT have been found to be mediated by increased growth factor synthesis, stem cell mobilization, attenuated monocyte chemokine production, neutrophil β-actin 5-nitrosylation, and changes in inflammatory markers, which overall lead to neovascularization and improved tissue survival [11,76,77]. Changes in oxygen transport and metabolism have also been implicated as mechanistically underlying HBOT. Recently, Li et al. conducted a proteomic analysis on HBOT in rats with spinal cord injuries and identified an upregulation in proteins involved in oxygen transport and binding [77]. Given that hypoxia is a key mediator of secondary injury in TBI, modulating oxygen transport may prove beneficial in TBI patients.

In the context of traumatic brain injury, an important therapeutic mechanism of interest is the inhibition of chronic neuroinflammation pathways. Chronic TBI, in which inflammation closely accompanies secondary brain injury, has been targeted by many potential therapeutics with an overall goal of preventing the further deficits and neurodegenerative processes [78,79]. Meng et al. found that in rats with post-trauma secondary brain injury, HBOT inhibits toll-like receptor (TLR)-4/nuclear factor kappa B (NF-kB) signaling and reduces the levels of multiple inflammatory and apoptotic markers including caspase-3, tumor necrosis factor α (TNF-α), interleukin (IL)-6, and IL-1β with overall reduced apoptosis and improved neurological functions [80]. Similarly, microglia contribute to the progression of secondary injury by producing inflammatory cytokines [81]. Lim et al. demonstrated a therapeutic anti-inflammatory effect of HBOT in rats 1 h and 8 h post-TBI. Specifically, they observed reduced microgliosis, neuronal apoptosis, and levels of TNF-α [82]. While these and other studies have shown that HBOT has therapeutic utility post-TBI, several studies have demonstrated a prophylactic potential of HBOT. Yang et al. demonstrated that HBOT preconditioning before ischemia attenuates inflammation by downregulating both allograft inflammatory factor (IBA)-1-positive microglia and levels of TNF-α [83]. Likewise, Lippert and Borlongan demonstrated that exposing rat neuronal cells to HBOT prior to the injection of TNF-a and lipopolysaccharide, both of which trigger a TBI-like inflammatory cascade, is cytoprotective. Specifically, cells pre-treated with HBOT had increased viability and higher levels of mitochondrial transfer from astrocytes [78]. Such an increased mitochondrial transfer may relate to the anti-inflammatory, anti-oxidative stress, and pro-stem cell proliferative effects of HBOT, though further prospective studies are warranted. These studies demonstrate both the therapeutic and prophylactic potential of HBOT for attenuating TBI-related brain injury (Table 2). 

## 5. Probing the Mechanisms Mediating HBOT via Biomolecular Assays

### 5.1. Inflammation (GFAP and UCH-L1)

Accumulating studies on TBI have shown sensitive biomarkers to determine the severity of the disease. Foremost are inflammatory biomarkers such as GFAP and UCH-L1 [84,85], which can be assayed in blood samples from TBI patients. For each sample draw, a single vial of approximately 2 mL of blood is collected and placed in serum separator tubes allowing it to clot at room temperature. The blood is then centrifuged for 30 min, the serum is placed in bar-coded aliquot containers, and stored in a freezer at −70 °C during its transportation to a central laboratory. There, the samples are analyzed in batches using sandwich enzyme-linked immunosorbent assays (ELISA) to GFAP [86,87,88,89] and UCH-L1 [90,91,92] (Abcam). Lab personnel running the samples stay blind to the clinical data. The necessary reagents, working standards, and samples are prepared and equilibrated at room temperature (18–25 °C) prior to use. Firstly, the wash buffer 1× by is diluted with 10× with deionized water. The antibody cocktail is mixed with the 10× capture and 10× detector antibodies with the antibody diluent keeping the dilution ratio of 1:1:8. Then, a fresh set of standards is prepared, assuring duplicate measurements for statistical reasons. The stock standard solution at 400 ng/mL is reconstituted with sample diluent and then prepared with an 8-point dilution series with the standard #8 as the blank control. Regarding samples, the cell culture supernatants are collected and centrifuged at 2000× *g* for 10 min to remove debris [93,94,95]. A 96-well plate is prepared with ready-to-use strips. For each assay performed, a minimum of two wells represents the zero control. A total of 50 µL of all samples or standards is added to appropriate wells. Subsequently, 50 µL of the antibody cocktail is added to each well. The loaded plate is placed on a plate shaker set to 400 rpm for 1 h of incubation at room temperature. At the end of the incubation period, three washes with 1× wash buffer into each well are performed, keeping the buffer for at least 10 s in the wells. After the last wash, an incubation of 10 min in the dark with 100 µL per each well of development solution occurs, setting the plate on the shaker at 400 rpm. The reaction is interrupted by the addition of 100 µL per well of stop solution. After 1 min on a plate shaker, the optical density at 450 nm is recorded in a plate reader as an endpoint reading [96].

### 5.2. Oxidative Stress (s100B, MBP, and NF-L)

In tandem with inflammatory biomarkers, a bulk of laboratory and clinical studies has also explored oxidative stress biomarkers such as s100B, MBP, and NF-L [97,98,99]. We also propose to obtain blood samples from TBI patients. For each sample drawn, a single vial of approximately 3 mL of blood is placed in serum separator tubes allowing it to clot at room temperature. The blood is centrifuged for 30 min, then the serum is placed in bar-coded aliquot containers, and stored in a freezer at −70 °C for transportation to a central laboratory. Thereafter, the samples are analyzed in batches using sandwich enzyme-linked immunosorbent assays (ELISA) to s100B [100,101,102], MBP [103,104,105] and NF–L [106,107,108] (R&D Systems). All reagents are prepared at room temperature. The capture antibody, biotinylated detection antibody and streptavidin conjugated to horseradish-peroxidase (Streptavidin-HRP B) are next processed following the working concentration specified on the vial label. In particular, the biotinylated detection antibody is diluted with 10 mL of reagent diluent. Then, Streptavidin-HRP B and biotinylated detection antibody are diluted with reagent diluent. The capture antibody is reconstituted with 0.5 mL of PBS and then diluted in PBS without a carrier protein. Subsequently, a seven-point standard curve is prepared using twofold serial dilutions in a reagent diluent. Each vial is reconstituted using standards with 0.5 mL of deionized water. Each plate assayed requires a total of 1000 μL of a high standard. All working dilutions are next prepared at the time of the assay. For the preparation of the assayed 96-wells microplate, the plate is coated with 100 μL per well of the diluted capture antibody. The coated microplate is sealed and incubated overnight at room temperature. After incubation, three washes are performed for each well using a squirt bottle and assuring the complete removal of the liquid at each step [109]. As the last wash occurs, any remaining wash buffer is removed by inverting the plate and blotting it against clean paper towels. The washed microplate will be blocked by non-specific sites by adding 300 μL of blocking buffer to each well. Then, the plate is incubated for a minimum of 1 h at room temperature. At the end of the incubation, three washes are performed assuring the complete removal of liquid at each step. Once the assayed microplate has its coating and blocking, it will be ready to receive samples and standards. A quantity of 100 μL of samples or standards is then added to the respective wells; the microplate is then sealed with an adhesive strip and incubated for 2 h at room temperature. After incubation, the samples and standards are processed with the microplate washes as described before, with at least three washes in the wash buffer. A quantity of 100 μL of diluted biotinylated detection antibody is added to each well. Again, the plate is sealed and incubated for 2 h at room temperature. After that, the biotinylated detection antibody is removed, and the plate is washed as described above. Next, 100 μL of diluted Streptavidin-HRP B are added to each well and the plate is incubated for 20 min at room temperature, while avoiding placing the plate in direct light. After three washes, 100 μL of the substrate solution are added to each well, then incubated for 20 min at room temperature, while avoiding exposure of the plate to direct light. The reaction is then interrupted and processed for the recording of the optical density immediately with a microplate reader set to 450 nm [110,111].

### 5.3. Mitochondria Activity (Seahorse)

Recent reports on TBI biomarker development have also highlighted the implication of mitochondrial activity in detecting disease onset and progression [112,113]. In particular, TBI patients have been shown to display altered circulating cell energy metabolism and respiration [112,113]. On the day of experiments, blood pellet or cells are detached from cell culture plates and seeded to a Seahorse 96-well plate (101085-004; Agilent, Santa Clara, CA, USA) [114,115]. The centrifugation method will immobilize the samples. Briefly, the Seahorse 96-well plate is centrifuged in a swing bucket rotator with slow acceleration (4 on a scale of 9) to a max speed of 450 rpm with 0 brake, and then the plate orientation reversed and centrifuged again to a max speed of 650 rpm with 0 brake. To determine the cellular oxygen consumption rate (OCR), we propose to use the Seahorse extracellular flux analyzer XFe96 (102416; Agilent) in combination with sequential injection of various compounds (1 µmol/L oligomycin, 1 µmol/L carbonyl cyanide 4-(trifluoromethoxy) phenylhydrazone (FCCP), and 0.5 µmol/L Rotenone and Antimycin A) [112,116]. The measurements of bioenergetics in platelets are performed using a Seahorse XFe96 Flux Analyzer (Agilent Technologies, Santa Clara, CA, USA). The OCR is measured in the presence of substrates, inhibitors, and uncouplers related to OXPHOS as modified from previously described methods [117,118]. The day before the planned experiment, the sensor cartridge of the extracellular flux kit is filled with 200 µL deionized water, kept at 37 °C overnight. At least 30 min prior to use, the water is removed, the plate filled with 200 µL XF calibrant solution, and the plate placed back at 37 °C. The loading of the injection ports, A to D of the sensor cartridge, separately or in combinations of substrates/inhibitors/uncouplers will allow measurement of different states of respiration. Before loading, the stocks are diluted in buffer such that after each sequential injection, the final concentration of the modulators is 5 mM pyruvate, 2.5 mM malate, and 10 mM succinate (via Port A), 5 µM oligomycin A (via Port B), 4 µM FCCP (via Port C), and 1 µM antimycin A (via Port D). Loading proceeds with either 10 or 30 µg total platelet protein (approximately 1 × 10^7^ or 3 × 10^7^ platelets) per well on a Seahorse XFe96 assay plate in a total volume of 30 µL (platelet sample plus buffer). The assay plates are next centrifuged at 2100× *g* for 4 min at RT. After that, a pre-warmed buffer is added to bring the starting volume to 175 µL. After the calibration step, the utility plate is placed onto the assay plate carrying platelet samples. The Seahorse Standard XFe96 flux assay plates are used for platelet analysis. The OCR and extra-cellular acidification rate (ECAR) are recorded in the absence or the presence of various substrates/inhibitors added from port A to port D [119]. Then, the measurements on intact (non-permeabilized) platelets are performed via basal readings in the presence of glucose in the buffer. The OXPHOS, LEAK, MAX, and non-mitochondrial oxygen consumption respiration rates are recorded depending on subsequent port injections. Basal glycolysis will reveal ECAR in the presence of glucose without substrate addition. Maximum glycolysis will reveal ECAR after the addition of oligomycin according to the injection paradigm [120]. The platelet coupling efficiency is calculated using OXPHOS/LEAK, while the glycolytic reserve capacity is analyzed by Max glycolysis-Basal glycolysis [121,122].

### 5.4. Stem Cell Proliferation (Flow Cytometry for Oct4, Nanog, and SSEA)

The most recent addition to TBI biomarker development involves characterizing circulating stem cells using proliferation markers such as Oct4, Nanog, and SSEA [123,124], which are established stem cell specific protein and gene markers [125]. The immunostaining of cells is performed using manufacturers’ recommended antibody dilution in staining buffer containing Hank’s balanced salt solution (Invitrogen, Carlsbad, CA, USA), 10% fetal bovine serum (Invitrogen) and 10 mM Hepes (Invitrogen). Flow cytometry sorting is conducted on FACS Aria II (BD Biosciences, Billerica, MA, USA) [126,127]. Cell sorting at clonal cell densities is carried out by sorting cells directly into 96-well plates. Briefly, the purified cells are prepared in a suitable isotonic neutrally buffered staining buffer in order to cushion damages during centrifugation, avoid non-specific staining, prevent capping of the bound antibody, and the block of Fc receptor binding. The concentration of the cell suspension will need to be of 5 × 10^5^ cells/mL, assuring by vital count a viability of more than 90%. The cells are then fixed and permeabilized in fix/perm buffer. For staining in microtiter plates, the plate is spun for 3 min at 100× *g* at 8 °C. Then, the plate is processed to resuspend the pellet immediately in 100 μL of primary antibodies for OCT4, Nanog, and SSEA markers diluted with the staining buffer [128,129,130]. The plate is then incubated for 30 min at 4 °C with the primary antibodies. At the end of the incubation period, the plate is washed three times in perm/wash buffer. After washing, the plate is immediately filled with 100 μL per well of secondary antibodies AlexaFluor 488 or 594 or 647. Again, the plate is washed in perm/wash buffer, resuspending the cells in staining buffer prior to analysis. Flow cytometry analysis is performed using Guava EasyCyte 8HT (Millipore, Lakeland, FL, USA) [131].

## 6. Efficacy of HBOT for Different TBI Populations

The use of HBOT for TBI patients has been controversial, but numerous studies have shown its benefits. Chen et al. conducted an extensive study and found that patients with moderate and severe TBI showed neurological improvements after HBOT at 2.0 ATA [132]. Consciousness recovery was measured using Coma Recovery Scale-Revised scores, which improved in TBI patients after HBOT [132]. Cognitive decline is a significant concern in TBI patients, and precipitates poor concentration, memory loss, and diminished executive ability [133]. Chen et al. also assessed the CBF and used Disability Rating Scale and Functional Independence Measure measurements, both of which indicated that HBOT improved short-term and long-term cognitive deficits in moderate and severe TBI [132]. Electroencephalogram (EEG) analysis revealed changes in δ and α wave activity, adding to a growing body of literature that suggests that HBOT has a positive impact on improving consciousness by reducing excessive slow waves associated with TBI [132,134]. Additionally, HBOT resulted in increased levels of the brain-derived neurotrophic factor (BDNF), the nerve growth factor (NGF), and the vascular endothelial growth factor (VEGF), indicating the potential for faster recovery and improved prognosis in TBI patients [132].

HBOT has been shown to help patients with specific TBI-related conditions, such as post-concussion syndrome and blast-related injuries. In one study, HBOT was postulated to help concussion-related injuries by allowing blood oxygen levels to penetrate deeper into the neural tissue, a feat that would be impossible without a hyperbaric chamber [135]. Specifically, HBOT appears to initiate cellular and vascular repair mechanisms and improves the cerebral vascular flow by acting on key targets such as injured neural fibers and axonal white matter [135,136]. In blast-related injuries, patients suffer cerebral vasculature changes that lead to microvasculature chronic disruptions long after the initial exposure event [137,138]. With HBOT, the hyper-oxygenated physiological condition can provide a neuroprotective or regenerative environment to promote angiogenesis and neurogenesis [138].

## 7. Safety of HBOT in TBI Populations

While infrequent, there have been adverse effects reported regarding the use of HBOT to treat TBI. These effects are attributed to the increase in atmospheric pressure, difficulty tolerating hyperbaric oxygen, and/or underlying psychological dysfunction [139]. Mild risks attributed to the change in atmospheric pressure include mild pressure-related tissue damage (barotrauma) or headaches [140]. If the pressure surpasses 2.4 ATA, however, patients are at increased risk of neurologic, pulmonary, and ocular oxygen toxicity [139]. Oxygen toxicity in these regions makes patients susceptible to seizures, loss of consciousness, oxidative stress, and temporary myopia (nearsightedness) [139]. Regarding psychological distress, some patients reported claustrophobia due to confinement during treatment [140].

Considering the variety of pathophysiologies underlying TBIs, the time and frequency of HBOT ought to vary based on patient tolerance and reaction to the conditions [140]. Breaking up exposure to hyperbaric oxygen into multiple sessions has resulted in higher tolerances and minimization of some adverse effects, such as pulmonary oxygen toxicity [141]. To combat mild ear barotrauma, patients were advised to relieve pressure via swallowing, chewing, or the Valsalva maneuver, a technique utilized to equalize ear pressure [139]. In cases where these techniques are not possible, such as for intubated patients, a myringotomy, an incision of the eardrum, may be performed to drain fluid from the inner ear and relieve pressure [139]. Furthermore, to maximize safety, there are medical professionals present during the duration of the treatment. The extent of effects and risks associated with HBOT can also be influenced by the classification of the TBI. When HBOT is utilized for chronic mTBI, adverse effects are at a minimum with the most prevalent being mild barotrauma [140]. However, when applied in moderate to severe acute TBI cases more severe consequences can occur such as pulmonary complications and seizures [140]. Such differences can be attributed to the circumstances in which HBOT is utilized. Patients with moderate to severe acute TBI typically require life-saving treatment due to the severity of their trauma. Therefore, this worsened condition has the potential to result in additional risk when the patient is subjected to HBOT [140]. 

## 8. Conclusions

Whether occurring as the result of a fall, gunshot wound, or any other noxious stimulus, TBIs often precede devastating consequences. On a cellular level, acute brain injuries result in neuronal and glial necrosis. However, secondary brain injuries are more insidious, and result in cellular morphological changes, loss of membrane integrity, excitotoxicity, and neuroinflammation. Though mTBIs may recover completely, these aforementioned chronic changes may precipitate crippling post-concussive symptoms, particularly in more severe TBIs. Of interest, then, are therapies to target the etiologies of secondary brain injuries. HBOT is utilized in multiple pathologies but has demonstrated promising results in the preclinical and clinical trial environment. HBOT ameliorates cerebral edema, intracranial pressure elevation, apoptosis, and neuroinflammation while increasing oxygen delivery to the brain and cerebral glucose utilization. Herein is a review of the existing clinical research examining the use of HBOT in attenuating both the symptoms and physical manifestations of TBI.

## Figures and Tables

**Table 1:** Clinical and pre-clinical trials evaluating HBOT in TBI patients. Most of the clinical trials utilized 1.5 ATM for 40 HBOT sessions (“dives”) and determined that while HBOT is safe for patients with mild, moderate, and severe TBI, it is only effective in moderate to severe TBI patients. In contrast, a few preclinical studies utilized 1.5 ATM and applied fewer dives to each TBI patient. These preclinical studies deemed HBOT as safe and effective in mild, moderate, and severe TBI patients and postulated that HBOT exerts its effects by modulating inflammation creating an anti-oxidative environment, repairing mitochondria, and stimulating stem cell proliferation.

Clinical and Pre-Clinical Studies on HBOT	
**Clinical Studies**	**Preclinical Studies**
1.5 atms, 40 dives	1.5 atms, few dives
Safe: Mild, Moderate, Severe TBI	Safe: Mild, Moderate, Severe TBI
Effective: Moderate-Severe TBI only	Effective: Mild, Moderate, Severe TBI
Mechanisms: Anti-inflammation Anti-oxidative stress Mitochondrial repair Stem cell proliferation	

**Table 2:** Current Clinical Studies on HBOT in TBI Populations. This table summarizes clinical trials investigating the use of HBOT in various TBI populations. TBI = traumatic brain injury, HBOT = hyperbaric oxygen therapy, PI = principal investigator, PPCS = persistent post-concussion syndrome, HBO2T = hyperbaric oxygen therapy, PCS = post-concussive symptoms, mTBI = mild traumatic brain injury, SPECT = single-photon emission computed tomography, HBO2 = hyperbaric oxygen therapy, MRI = magnetic resonance imaging, DTI = diffusion tensor imaging.

Trial	Status	Trial Details (PI, Main Country)	Population	Primary Outcome	Primary Outcome Measurement	Primary Results	Treatment
The Role of Hyperbaric Oxygen and Neuropsychological Therapy in Cognitive Function Following TBI	Terminated	Dr. Tsang-Tang Hsieh, Taiwan	Mild and moderate TBI	Neuropsychological function	Neuropsychological evaluation	Terminated due to the COVID-19 pandemic	30 HBOT sessions, 60 min each
HBOT to Treat Mild TBI/PPCS	Completed	Dr. Paul G. Harch, United States of America	Mild TBI	Working memory Neurobehavioral symptoms	Neuropsychological evaluation Neuro-behavioral symptom inventory	HBOT group showed persistent improvement in outcomes [37]	40 HBOT sessions, 45 min each
HBO2T for PCS After mTBI	Completed	Dr. David X. Cifu & Dr. Brett Hart, United States of America	Mild TBI	Post-concussive symptoms	Symptom assessment Battery for post-concussive symptoms	There was no significant difference in PCS between the sham and HBOT groups [38,39,40,41,42,43]	40 HBOT sessions, 60 min each
Hyperbaric Treatment of TBI	Completed	Dr. Barry Miskin, United States of America	All TBI stages with loss of consciousness	Cerebral blood flow	Single-photon emission Computerized tomography (SPECT) imaging	Missing	120 HBOT sessions, 60 min each
Hyperbaric Oxygen for Traumatic and Non-Traumatic Brain Injury	Completed	Dr. Lindell K. Weaver, United States of America	Symptomatic brain injury	Neurobehavioral symptoms	Reported symptoms	HBOT was associated with improved sleep quality, PCS, PTSD, and cognition but not eye movements [44,45,46]	40 HBOT sessions, 60 min each
Hyperbaric Oxygen Therapy and SPECT Brain Imaging in TBI	Unknown	Dr. Paul G. Harch, United States of America	All TBI stages	Cerebral blood flow	SPECT imaging	Missing	HBOT sessions
Hyperbaric Oxygen Brain Injury Treatment Trial (HOBIT)	Recruiting	Dr. Gaylan L Rockswold, Dr. William Barsan, Dr. Byron Gajewski, Dr. Frederick K. Korley, United States of America	Severe TBI	Consciousness	Glasgow Outcome Scale extended	Actively recruiting	Up to 10 HBOT sessions, 60 min each
HBO2 for Persistent Post-concussive Symptoms after mTBI (HOPPS)	Completed	Dr. Scott Miller, United States of America	Mild TBI	Post-concussive symptoms	Rivermead post-concussion symptom questionnaire	HBO2 did not produce a benefit in PCS and was not related to serious adverse events [47,48,49]	40 HBOT sessions, 60 min each
Comparison Between Different Types of Oxygen Treatment Following TBI	Completed	Dr. Gary L. Rockswold, United States of America	Mild or moderate brain injury with acute deterioration	Cerebral metabolic rate of oxygen (CMRO2) Microdialysis lactate Brain tissue oxygen (PtO2) Intracranial pressure (ICP)	Missing	Missing	HBOT
The Effect of HBOT on Patients Suffering From Neurologic Deficiency Due to TBI	Completed	Missing, Israel	Mild TBI	Neurological function	Neurocognitive assessment	Missing	40 HBOT sessions, 60 min each
Treatment of TBI with HBOT	Completed	Dr. Leonardo C. Profenna, United States of America	Mild to moderate TBI	Cognitive function PTSD symptoms	Neurocognitive assessment PTSD checklist	HBO2 did not produce a benefit in PCS and was not related to serious adverse events [43,50]	30 HBOT sessions, 3 30-min sessions each
Cognitive Profile of Patients at the Sagol Center for Hyperbaric Medicine and Research	Active, Not Recruiting	Missing, Israel	Chronic brain injury or cognitive impairment	Cognitive function	Neurocognitive assessment	Active study	40–60 HBOT sessions, 90 min each
HBOT in Chronic TBI/PCS and TBI/PTSD	Completed	Dr. Paul G. Harch, United States of America	Mild to moderate TBI	Cognitive function	Psychometric testing	HBO2 had improved cognitive function when compared to sham [51]	40 or 80 HBOT sessions, 60 min each
HBOT Effect on PCS in Children (TBIPED)	Terminated	Missing, Israel	Mild TBI	Cognitive function	Neurocognitive assessment (Neurotrax battery test)	Terminated due to recruitment issues (refusal to undergo sham controlled)	60 HBOT sessions, 60 min each
The Effects of Hyperbaric Oxygen on Non-Acute TBI	Recruiting	Dr. Bing Xiong, China	Moderate and severe TBI	Consciousness Cognitive impairment	Glasgow Outcome Scale Disability rating scale	Actively recruiting	Missing
The Biomarkers in the Hyperbaric Oxygen Brain Injury Treatment Trial (BIOHOBIT)	Recruiting	Dr. Frederick Korley, United States of America	Severe TBI	Neurological status	Glasgow Outcome Scale	Actively recruiting	Missing
Test of Chamber Pressure on Divers and Chamber Attendants (TOP-DIVER)	Completed	Dr. Lindell K. Weaver, United States of America	Scuba divers	Perception of depth Perception of breathing gas	Questionnaires	Experienced divers are unable to differentiate between 1.2 and 1.5 atm [52]	Missing
MRI Brain Changes Induced by HBOT in Brain Injury Patients	Unknown	Missing, Israel	Chronic TBI	Cerebral perfusion White matter microstructure	Perfusion MRI DTI	Missing	Missing
Hyperbaric Oxygen Effects on Persistent Post-Concussive Symptoms (HOINPCS)	Recruiting	Dr. Olayinka D. Ajayi, Dr. Marc Basson, United States of America	Mild TBI	Neuropsychological function	Neuropsychological assessment	Actively recruiting	40 HBOT sessions, 60 min each
HBOT in Chronic TBI or PTSD (NBIRR-1)	Terminated	Dr. Robert Mozayeni, United States of America	Mild to moderate TBI	Cognitive function	Missing	Terminated due to new regulatory requirements	Missing
mTBI Mechanisms of Action of HBO2 for Persistent Post-Concussive Symptoms (BIMA)	Completed	Dr. Lindell Weaver, United States of America	Mild TBI	Adverse events	Safety evaluations	HBOT was not associated with serious adverse effects [45,47,49,53,54,55,56,57,58,59,60,61]	40 HBOT sessions
Normative Datasets for Assessments Planned for Mild Traumatic Brain Injury (NORMAL)	Completed	Dr. Robert C. Price, United States of America	Mild TBI	Neuropsychological function	Neuropsychological assessments	Missing	Missing
Hyperbaric Oxygen Treatment to Treat mTBI/PPCS	Unknown	Dr. Paul G. Harch, United States of America	Mild TBI	Neuropsychological function Neurobehavioral symptoms	Missing	Missing	40 HBOT sessions, 45 min each
HBOT for PCS	Unknown	Dr. David W. Harrison, Canada	PCS	Post-concussion symptoms	Post-concussion symptoms questionnaire	Missing	Missing number of sessions, 90 min each
Hyperbaric Oxygen Therapy for Post-Concussion Syndrome	Recruiting	Dr. Shanti Pino, United States of America	Mild TBI	Post-concussion symptoms	Post-concussion symptoms questionnaire	Actively recruiting	20 HBOT sessions, 90 min each
HBO2 for Post-concussive Symptoms after mTBI (HOPPS)	Completed	Dr. Scott Miller, United States of America	Mild TBI	Post-concussion symptoms	Post-concussion symptoms questionnaire	PCS between HBO2 and sham did not significantly differ [47,48,49]	40 HBOT sessions, 60 min each

## Data Availability

Not applicable.

## Abbreviations

TBI	Traumatic brain injury
GCS	Glasgow Coma Scale
HBOT	Hyperbaric oxygen therapy
ROS	Reactive oxygen species
RNS	Reactive nitrogen species
ICP	Intracranial pressure
GOS	Glasgow outcome scale
mTBI	Mild traumatic brain injury
PPCS	Persistent post-concussion symptoms
CTE	Chronic traumatic encephalopathy
CBF	Cerebral blood flow
TLR	Toll like receptor
NFKB	Nuclear factor kappa B
TNF-α	Tumor necrosis factor A
IL	Interleukin
IBA	Allograft inflammatory factor
EEG	Electroencephalogram
BDNF	Brain-derived neurotrophic factor
NGF	Nerve growth factor
VEGF	Vascular endothelial growth factor

## References

[1] Menon D.K., Schwab K., Wright D.W., Maas A.I. (2010). Position statement: Definition of traumatic brain injury. Arch. Phys. Med. Rehabil..

[2] Kazim S.F., Shamim M.S., Tahir M.Z., Enam S.A., Waheed S. (2011). Management of penetrating brain injury. J. Emergencies Trauma Shock.

[3] Wolf S.J., Bebarta V.S., Bonnett C.J., Pons P.T., Cantrill S.V. (2009). Blast injuries. Lancet.

[4] Kinoshita K. (2016). Traumatic brain injury: Pathophysiology for neurocritical care. J. Intensive Care.

[5] Saatman K.E., Duhaime A.C., Bullock R., Maas A.I., Valadka A., Manley G.T. (2008). Classification of traumatic brain injury for targeted therapies. J. Neurotrauma.

[6] Teasdale G., Jennett B. (1974). Assessment of coma and impaired consciousness. A practical scale. Lancet.

[7] Teasdale G., Maas A., Lecky F., Manley G., Stocchetti N., Murray G. (2014). The Glasgow Coma Scale at 40 years: Standing the test of time. Lancet Neurol..

[8] McCrory P., Meeuwisse W.H., Aubry M., Cantu B., Dvorák J., Echemendia R.J., Engebretsen L., Johnston K., Kutcher J.S., Raftery M. (2013). Consensus statement on concussion in sport: The 4th International Conference on Concussion in Sport held in Zurich, November 2012. Br. J. Sports Med..

[9] Einarsen C.E., van der Naalt J., Jacobs B., Follestad T., Moen K.G., Vik A., Håberg A.K., Skandsen T. (2018). Moderate Traumatic Brain Injury: Clinical Characteristics and a Prognostic Model of 12-Month Outcome. World Neurosurg..

[10] Prabhakaran K., Petrone P., Lombardo G., Stoller C., Policastro A., Marini C.P. (2017). Mortality rates of severe traumatic brain injury patients: Impact of direct versus nondirect transfers. J. Surg. Res..

[11] Thom S.R. (2011). Hyperbaric oxygen: Its mechanisms and efficacy. Plast. Reconstr. Surg..

[12] Guedes V.A., Song S., Provenzano M., Borlongan C.V. (2016). Understanding the pathology and treatment of traumatic brain injury and posttraumatic stress disorder: A therapeutic role for hyperbaric oxygen therapy. Expert Rev. Neurother..

[13] Ikeda Y., Long D.M. (1990). The molecular basis of brain injury and brain edema: The role of oxygen free radicals. Neurosurgery.

[14] Coe J.E., Hayes T.M. (1966). Treatment of experimental brain injury by hyperbaric oxygenation. Preliminary report. Am. Surg..

[15] Dunn J., Lawson D. (1966). Effects of Hypobaric and Hyperbaric Oxygen on Experimental brain injury. Orig. Hyperb. Med..

[16] Miller J.D., Fitch W., Ledingham I.M., Jennett W.B. (1970). The effect of hyperbaric oxygen on experimentally increased intracranial pressure. J. Neurosurg..

[17] Palzur E., Vlodavsky E., Mulla H., Arieli R., Feinsod M., Soustiel J.F. (2004). Hyperbaric oxygen therapy for reduction of secondary brain damage in head injury: An animal model of brain contusion. J. Neurotrauma.

[18] Palzur E., Zaaroor M., Vlodavsky E., Milman F., Soustiel J.F. (2008). Neuroprotective effect of hyperbaric oxygen therapy in brain injury is mediated by preservation of mitochondrial membrane properties. Brain Res..

[19] Vlodavsky E., Palzur E., Soustiel J.F. (2006). Hyperbaric oxygen therapy reduces neuroinflammation and expression of matrix metalloproteinase-9 in the rat model of traumatic brain injury. Neuropathol. Appl. Neurobiol..

[20] Chen X., Duan X.S., Xu L.J., Zhao J.J., She Z.F., Chen W.W., Zheng Z.J., Jiang G.D. (2014). Interleukin-10 mediates the neuroprotection of hyperbaric oxygen therapy against traumatic brain injury in mice. Neuroscience.

[21] Daugherty W.P., Levasseur J.E., Sun D., Rockswold G.L., Bullock M.R. (2004). Effects of hyperbaric oxygen therapy on cerebral oxygenation and mitochondrial function following moderate lateral fluid-percussion injury in rats. J. Neurosurg..

[22] Contreras F.L., Kadekaro M., Eisenberg H.M. (1988). The effect of hyperbaric oxygen on glucose utilization in a freeze-traumatized rat brain. J. Neurosurg..

[23] Brkic P., Stojiljkovic M., Jovanovic T., Dacic S., Lavrnja I., Savic D., Parabucki A., Bjelobaba I., Rakic L., Pekovic S. (2012). Hyperbaric oxygenation improves locomotor ability by enhancing neuroplastic responses after cortical ablation in rats. Brain Inj..

[24] Niklas A., Brock D., Schober R., Schulz A., Schneider D. (2004). Continuous measurements of cerebral tissue oxygen pressure during hyperbaric oxygenation--HBO effects on brain edema and necrosis after severe brain trauma in rabbits. J. Neurol. Sci..

[25] Lin K.C., Niu K.C., Tsai K.J., Kuo J.R., Wang L.C., Chio C.C., Chang C.P. (2012). Attenuating inflammation but stimulating both angiogenesis and neurogenesis using hyperbaric oxygen in rats with traumatic brain injury. J. Trauma Acute Care Surg..

[26] Harch P.G., Kriedt C., Van Meter K.W., Sutherland R.J. (2007). Hyperbaric oxygen therapy improves spatial learning and memory in a rat model of chronic traumatic brain injury. Brain Res..

[27] Hardy P., Johnston K.M., De Beaumont L., Montgomery D.L., Lecomte J.M., Soucy J.P., Bourbonnais D., Lassonde M. (2007). Pilot case study of the therapeutic potential of hyperbaric oxygen therapy on chronic brain injury. J. Neurol. Sci..

[28] Hu Q., Manaenko A., Xu T., Guo Z., Tang J., Zhang J.H. (2016). Hyperbaric oxygen therapy for traumatic brain injury: Bench-to-bedside. Med. Gas Res..

[29] Wang G.H., Zhang X.G., Jiang Z.L., Li X., Peng L.L., Li Y.C., Wang Y. (2010). Neuroprotective effects of hyperbaric oxygen treatment on traumatic brain injury in the rat. J. Neurotrauma.

[30] Rockswold S.B., Rockswold G.L., Vargo J.M., Erickson C.A., Sutton R.L., Bergman T.A., Biros M.H. (2001). Effects of hyperbaric oxygenation therapy on cerebral metabolism and intracranial pressure in severely brain injured patients. J. Neurosurg..

[31] Sukoff M.H., Ragatz R.E. (1982). Hyperbaric oxygenation for the treatment of acute cerebral edema. Neurosurgery.

[32] Lin J.W., Tsai J.T., Lee L.M., Lin C.M., Hung C.C., Hung K.S., Chen W.Y., Wei L., Ko C.P., Su Y.K. (2008). Effect of hyperbaric oxygen on patients with traumatic brain injury. Acta Neurochir. Suppl..

[33] Prakash A., Parelkar S.V., Oak S.N., Gupta R.K., Sanghvi B.V., Bachani M., Patil R. (2012). Role of hyperbaric oxygen therapy in severe head injury in children. J. Pediatr. Neurosci..

[34] Holbach K.H., Wassmann H., Kolberg T. (1974). Improved reversibility of the traumatic midbrain syndrome using hyperbaric oxygen. Acta Neurochir..

[35] Artru F., Chacornac R., Deleuze R. (1976). Hyperbaric oxygenation for severe head injuries. Preliminary results of a controlled study. Eur. Neurol..

[36] Rockswold G.L., Ford S.E., Anderson D.C., Bergman T.A., Sherman R.E. (1992). Results of a prospective randomized trial for treatment of severely brain-injured patients with hyperbaric oxygen. J. Neurosurg..

[37] Harch P.G., Andrews S.R., Rowe C.J., Lischka J.R., Townsend M.H., Yu Q., Mercante D.E. (2020). Hyperbaric oxygen therapy for mild traumatic brain injury persistent postconcussion syndrome: A randomized controlled trial. Med. Gas Res..

[38] Cifu D.X., Hart B.B., West S.L., Walker W., Carne W. (2014). The effect of hyperbaric oxygen on persistent postconcussion symptoms. J. Head Trauma Rehabil..

[39] Cifu D.X., Hoke K.W., Wetzel P.A., Wares J.R., Gitchel G., Carne W. (2014). Effects of hyperbaric oxygen on eye tracking abnormalities in males after mild traumatic brain injury. J. Rehabil. Res. Dev..

[40] Cifu D.X., Walker W.C., West S.L., Hart B.B., Franke L.M., Sima A., Graham C.W., Carne W. (2014). Hyperbaric oxygen for blast-related postconcussion syndrome: Three-month outcomes. Ann. Neurol..

[41] Walker W.C., Franke L.M., Cifu D.X., Hart B.B. (2014). Randomized, Sham-Controlled, Feasibility Trial of Hyperbaric Oxygen for Service Members with Postconcussion Syndrome: Cognitive and Psychomotor Outcomes 1 Week Postintervention. Neurorehabilit. Neural Repair.

[42] Wolf G., Cifu D., Baugh L., Carne W., Profenna L. (2012). The effect of hyperbaric oxygen on symptoms after mild traumatic brain injury. J. Neurotrauma.

[43] DeCato T.W., Bradley S.M., Wilson E.L., Harlan N.P., Villela M.A., Weaver L.K., Hegewald M.J. (2019). Effects of sprint interval training on cardiorespiratory fitness while in a hyperbaric oxygen environment. Undersea Hyperb. Med..

[44] Walker J.M., Mulatya C., Hebert D., Wilson S.H., Lindblad A.S., Weaver L.K. (2018). Sleep assessment in a randomized trial of hyperbaric oxygen in U.S. service members with post concussive mild traumatic brain injury compared to normal controls. Sleep Med..

[45] Weaver L.K., Wilson S.H., Lindblad A.S., Churchill S., Deru K., Price R.C., Williams C.S., Orrison W.W., Walker J.M., Meehan A. (2018). Hyperbaric oxygen for post-concussive symptoms in United States military service members: A randomized clinical trial. Undersea Hyperb. Med..

[46] Churchill S., Deru K., Weaver L.K., Wilson S.H., Hebert D., Miller R.S., Lindblad A.S. (2019). Adverse events and blinding in two randomized trials of hyperbaric oxygen for persistent post-concussive symptoms. Undersea Hyperb. Med..

[47] Miller R.S., Weaver L.K., Bahraini N., Churchill S., Price R.C., Skiba V., Caviness J., Mooney S., Hetzell B., Liu J. (2015). Effects of hyperbaric oxygen on symptoms and quality of life among service members with persistent postconcussion symptoms: A randomized clinical trial. JAMA Intern. Med..

[48] Weaver L.K., Churchill S., Wilson S.H., Hebert D., Deru K., Lindblad A.S. (2019). A composite outcome for mild traumatic brain injury in trials of hyperbaric oxygen. Undersea Hyperb. Med..

[49] Wolf E.G., Prye J., Michaelson R., Brower G., Profenna L., Boneta O. (2012). Hyperbaric side effects in a traumatic brain injury randomized clinical trial. Undersea Hyperb. Med..

[50] Harch P.G., Andrews S.R., Fogarty E.F., Amen D., Pezzullo J.C., Lucarini J., Aubrey C., Taylor D.V., Staab P.K., Van Meter K.W. (2012). A phase I study of low-pressure hyperbaric oxygen therapy for blast-induced post-concussion syndrome and post-traumatic stress disorder. J. Neurotrauma.

[51] Weaver L.K., Churchill S.K., Bell J., Deru K., Snow G.L. (2012). A blinded trial to investigate whether ‘pressure-familiar’ individuals can determine chamber pressure. Undersea Hyperb. Med..

[52] Hart B.B., Wilson S.H., Churchill S., Deru K., Weaver L.K., Minnakanti M., Lindblad A.S. (2019). Extended follow-up in a randomized trial of hyperbaric oxygen for persistent post-concussive symptoms. Undersea Hyperb. Med..

[53] Wetzel P.A., Lindblad A.S., Mulatya C., Kannan M.A., Villmar Z., Gitchel G.T., Weaver L.K. (2019). Eye tracker outcomes in a randomized trial of 40 sessions of hyperbaric oxygen or sham in participants with persistent post concussive symptoms. Undersea Hyperb. Med..

[54] Wetzel P.A., Lindblad A.S., Raizada H., James N., Mulatya C., Kannan M.A., Villamar Z., Gitchel G.T., Weaver L.K. (2018). Eye Tracking Results in Postconcussive Syndrome Versus Normative Participants. Investig. Ophthalmol. Vis. Sci..

[55] Meehan A., Searing E., Weaver L.K., Lewandowski A. (2016). Baseline vestibular and auditory findings in a trial of post-concussive syndrome. Undersea Hyperb. Med..

[56] Wilson S.H., Weaver L.K., Lindblad A.S. (2016). Neuropsychological assessments in a hyperbaric trial of post-concussive symptoms. Undersea Hyperb. Med..

[57] Walker J.M., James N.T., Campbell H., Wilson S.H., Churchill S., Weaver L.K. (2016). Sleep assessments for a mild traumatic brain injury trial in a military population. Undersea Hyperb. Med..

[58] Mirow S., Wilson S.H., Weaver L.K., Churchill S., Deru K., Lindblad A.S. (2016). Linear analysis of heart rate variability in post-concussive syndrome. Undersea Hyperb. Med..

[59] Williams C.S., Spitz M.C., Foley J.F., Weaver L.K., Lindblad A.S., Wierzbicki M.R. (2016). Baseline EEG abnormalities in mild traumatic brain injury from the BIMA study. Undersea Hyperb. Med..

[60] Williams C.S., Weaver L.K., Lindblad A.S., Kumar S., Langford D.R. (2016). Baseline neurological evaluations in a hyperbaric trial of post-concussive syndrome. Undersea Hyperb. Med..

[61] Weaver L.K., Chhoeu A., Lindblad A.S., Churchill S., Wilson S.H. (2016). Hyperbaric oxygen for mild traumatic brain injury: Design and baseline summary. Undersea Hyperb. Med..

[62] Laskowski R.A., Creed J.A., Raghupathi R., Kobeissy F.H. (2015). Pathophysiology of Mild TBI: Implications for Altered Signaling Pathways. Brain Neurotrauma: Molecular, Neuropsychological, and Rehabilitation Aspects.

[63] Tenovuo O., Diaz-Arrastia R., Goldstein L.E., Sharp D.J., van der Naalt J., Zasler N.D. (2021). Assessing the Severity of Traumatic Brain Injury-Time for a Change?. J. Clin. Med..

[64] Jurick S.M., Crocker L.D., Merritt V.C., Sanderson-Cimino M.E., Keller A.V., Glassman L.H., Twamley E.W., Rodgers C.S., Schiehser D.M., Aupperle R.L. (2021). Independent and Synergistic Associations Between TBI Characteristics and PTSD Symptom Clusters on Cognitive Performance and Postconcussive Symptoms in Iraq and Afghanistan Veterans. J. Neuropsychiatry Clin. Neurosci..

[65] Howlett J.R., Nelson L.D., Stein M.B. (2022). Mental Health Consequences of Traumatic Brain Injury. Biol. Psychiatry.

[66] Madsen B.A., Fure S.C.R., Andelic N., Loke D., Lovstad M., Roe C., Howe E.I. (2023). Exploring the Association between Personality Traits, Symptom Burden, and Return to Work after Mild-to-Moderate Traumatic Brain Injury. J. Clin. Med..

[67] Jaganathan K.S., Sullivan K.A. (2020). Moving towards individualised and interdisciplinary approaches to treat persistent post-concussion symptoms. eClinicalMedicine.

[68] Goldstein L.E., Fisher A.M., Tagge C.A., Zhang X.L., Velisek L., Sullivan J.A., Upreti C., Kracht J.M., Ericsson M., Wojnarowicz M.W. (2012). Chronic traumatic encephalopathy in blast-exposed military veterans and a blast neurotrauma mouse model. Sci. Transl. Med..

[69] McKee A.C., Daneshvar D.H. (2015). The neuropathology of traumatic brain injury. Handb. Clin. Neurol..

[70] Ng S.Y., Lee A.Y.W. (2019). Traumatic Brain Injuries: Pathophysiology and Potential Therapeutic Targets. Front. Cell. Neurosci..

[71] Monsour M., Ebedes D., Borlongan C.V. (2022). A review of the pathology and treatment of TBI and PTSD. Exp. Neurol..

[72] Fehily B., Fitzgerald M. (2017). Repeated Mild Traumatic Brain Injury: Potential Mechanisms of Damage. Cell Transplant..

[73] Patel N.A., Moss L.D., Lee J.Y., Tajiri N., Acosta S., Hudson C., Parag S., Cooper D.R., Borlongan C.V., Bickford P.C. (2018). Long noncoding RNA MALAT1 in exosomes drives regenerative function and modulates inflammation-linked networks following traumatic brain injury. J. Neuroinflammation.

[74] Zhang R., Liu Y., Yan K., Chen L., Chen X.R., Li P., Chen F.F., Jiang X.D. (2013). Anti-inflammatory and immunomodulatory mechanisms of mesenchymal stem cell transplantation in experimental traumatic brain injury. J. Neuroinflammation.

[75] Camandola S., Mattson M.P. (2017). Brain metabolism in health, aging, and neurodegeneration. EMBO J..

[76] Casanova-Maldonado I., Arancibia D., Lois P., Pena-Villalobos I., Palma V. (2023). Hyperbaric oxygen treatment increases intestinal stem cell proliferation through the mTORC1/S6K1 signaling pathway in Mus musculus. Biol. Res..

[77] Li Z., Hou X., Liu X., Ma L., Tan J. (2022). Hyperbaric Oxygen Therapy-Induced Molecular and Pathway Changes in a Rat Model of Spinal Cord Injury: A Proteomic Analysis. Dose Response.

[78] Lippert T., Borlongan C.V. (2019). Prophylactic treatment of hyperbaric oxygen treatment mitigates inflammatory response via mitochondria transfer. CNS Neurosci. Ther..

[79] Zhai W.W., Sun L., Yu Z.Q., Chen G. (2016). Hyperbaric oxygen therapy in experimental and clinical stroke. Med. Gas Res..

[80] Meng X.E., Zhang Y., Li N., Fan D.F., Yang C., Li H., Guo D.Z., Pan S.Y. (2016). Hyperbaric Oxygen Alleviates Secondary Brain Injury After Trauma through Inhibition of TLR4/NF-kappaB Signaling Pathway. Med. Sci. Monit..

[81] Donat C.K., Scott G., Gentleman S.M., Sastre M. (2017). Microglial Activation in Traumatic Brain Injury. Front. Aging Neurosci..

[82] Lim S.W., Wang C.C., Wang Y.H., Chio C.C., Niu K.C., Kuo J.R. (2013). Microglial activation induced by traumatic brain injury is suppressed by postinjury treatment with hyperbaric oxygen therapy. J. Surg. Res..

[83] Yang L., Tang J., Chen Q., Jiang B., Zhang B., Tao Y., Li L., Chen Z., Zhu G. (2015). Hyperbaric oxygen preconditioning attenuates neuroinflammation after intracerebral hemorrhage in rats by regulating microglia characteristics. Brain Res..

[84] Papa L., Brophy G.M., Alvarez W., Hirschl R., Cress M., Weber K., Giordano P. (2023). Sex Differences in Time Course and Diagnostic Accuracy of GFAP and UCH-L1 in Trauma Patients with Mild Traumatic Brain Injury. Sci. Rep..

[85] Lagares A., Payen J.-F., Biberthaler P., Poca M.A., Méjan O., Pavlov V., Viglino D., Sapin V., Lassaletta A., de la Cruz J. (2023). Study Protocol for Investigating the Clinical Performance of an Automated Blood Test for Glial Fibrillary Acidic Protein and Ubiquitin Carboxy-Terminal Hydrolase L1 Blood Concentrations in Elderly Patients with Mild Traumatic BRAIN Injury and Reference Values (BRAINI-2 Elderly European Study): A Prospective Multicentre Observational Study. BMJ Open.

[86] Okonkwo D.O., Yue J.K., Puccio A.M., Panczykowski D.M., Inoue T., McMahon P.J., Sorani M.D., Yuh E.L., Lingsma H.F., Maas A.I.R. (2013). GFAP-BDP as an Acute Diagnostic Marker in Traumatic Brain Injury: Results from the Prospective Transforming Research and Clinical Knowledge in Traumatic Brain Injury Study. J. Neurotrauma.

[87] Lei J., Gao G., Feng J., Jin Y., Wang C., Mao Q., Jiang J. (2015). Glial Fibrillary Acidic Protein as a Biomarker in Severe Traumatic Brain Injury Patients: A Prospective Cohort Study. Crit. Care.

[88] Lee J.Y., Lee C.Y., Kim H.R., Lee C.-H., Kim H.W., Kim J.H. (2015). A Role of Serum-Based Neuronal and Glial Markers as Potential Predictors for Distinguishing Severity and Related Outcomes in Traumatic Brain Injury. J. Korean Neurosurg. Soc..

[89] Plog B.A., Dashnaw M.L., Hitomi E., Peng W., Liao Y., Lou N., Deane R., Nedergaard M. (2015). Biomarkers of Traumatic Injury Are Transported from Brain to Blood via the Glymphatic System. J. Neurosci..

[90] Papa L., Lewis L.M., Silvestri S., Falk J.L., Giordano P., Brophy G.M., Demery J.A., Liu M.C., Mo J., Akinyi L. (2012). Serum Levels of Ubiquitin C-Terminal Hydrolase (UCH-L1) Distinguish Mild Traumatic Brain Injury (TBI) from Trauma Controls and Are Elevated in Mild and Moderate TBI Patients with Intracranial Lesions and Neurosurgical Intervention. J. Trauma Acute Care Surg..

[91] Brophy G.M., Mondello S., Papa L., Robicsek S.A., Gabrielli A., Tepas J., Buki A., Robertson C., Tortella F.C., Hayes R.L. (2011). Biokinetic Analysis of Ubiquitin C-Terminal Hydrolase-L1 (UCH-L1) in Severe Traumatic Brain Injury Patient Biofluids. J. Neurotrauma.

[92] Mondello S., Kobeissy F., Vestri A., Hayes R.L., Kochanek P.M., Berger R.P. (2016). Serum Concentrations of Ubiquitin C-Terminal Hydrolase-L1 and Glial Fibrillary Acidic Protein after Pediatric Traumatic Brain Injury. Sci. Rep..

[93] Petzold A., Keir G., Green A.J.E., Giovannoni G., Thompson E.J. (2004). An ELISA for Glial Fibrillary Acidic Protein. J. Immunol. Methods.

[94] Zoltewicz J.S., Scharf D., Yang B., Chawla A., Newsom K.J., Fang L. (2012). Characterization of Antibodies that Detect Human GFAP after Traumatic Brain Injury. Biomark. Insights.

[95] Lindblad C., Pin E., Just D., Al Nimer F., Nilsson P., Bellander B.-M., Svensson M., Piehl F., Thelin E.P. (2021). Fluid Proteomics of CSF and Serum Reveal Important Neuroinflammatory Proteins in Blood–Brain Barrier Disruption and Outcome Prediction Following Severe Traumatic Brain Injury: A Prospective, Observational Study. Crit. Care.

[96] O’Connell G.C., Alder M.L., Smothers C.G., Still C.H., Webel A.R., Moore S.M. (2020). Use of High-Sensitivity Digital ELISA Improves the Diagnostic Performance of Circulating Brain-Specific Proteins for Detection of Traumatic Brain Injury during Triage. Neurol. Res..

[97] Biberthaler P., Linsenmeier U., Pfeifer K.-J., Kroetz M., Mussack T., Kanz K.-G., Hoecherl E.F.J., Jonas F., Marzi I., Leucht P. (2006). Serum S-100b Concentration Provides Additional Information Fot the Indication of Computed Tomography in Patients after Minor Head Injury: A Prospective Multicenter Study. Shock.

[98] Yamazaki Y., Yada K., Morii S., Kitahara T., Ohwada T. (1995). Diagnostic Significance of Serum Neuron-Specific Enolase and Myelin Basic Protein Assay in Patients with Acute Head Injury. Surg. Neurol..

[99] Shahim P., Politis A., van der Merwe A., Moore B., Chou Y.-Y., Pham D.L., Butman J.A., Diaz-Arrastia R., Gill J.M., Brody D.L. (2020). Neurofilament Light as a Biomarker in Traumatic Brain Injury. Neurology.

[100] Goyal A., Failla M.D., Niyonkuru C., Amin K., Fabio A., Berger R.P., Wagner A.K. (2013). S100b as a Prognostic Biomarker in Outcome Prediction for Patients with Severe Traumatic Brain Injury. J. Neurotrauma.

[101] Abbasi M., Sajjadi M., Fathi M., Maghsoudi M. (2014). Serum S100B Protein as an Outcome Prediction Tool in Emergency Department Patients with Traumatic Brain Injury. Turk. J. Emerg. Med..

[102] Borg K., Bonomo J., Jauch E.C., Kupchak P., Stanton E.B., Sawadsky B. (2012). Serum Levels of Biochemical Markers of Traumatic Brain Injury. Int. Sch. Res. Not..

[103] Wąsik N., Sokół B., Hołysz M., Mańko W., Juszkat R., Jagodziński P.P., Jankowski R. (2020). Serum Myelin Basic Protein as a Marker of Brain Injury in Aneurysmal Subarachnoid Haemorrhage. Acta Neurochir..

[104] Singh A., Singh K., Sahu A., Prasad R.S., Pandey N., Dhar S. (2022). Serum Concentration of Myelin Basic Protein as a Prognostic Marker in Mild-to-Moderate Head Injury Patients: A Prospective Study in a Tertiary Care Center. Indian. J. Neurosurg..

[105] Shang Y., Wang Y., Guo Y., Ren L., Zhang X., Wang S., Zhang C., Cai J. (2022). Analysis of the Risk of Traumatic Brain Injury and Evaluation Neurogranin and Myelin Basic Protein as Potential Biomarkers of Traumatic Brain Injury in Postmortem Examination. Forensic Sci. Med. Pathol..

[106] Nimer F.A., Thelin E., Nyström H., Dring A.M., Svenningsson A., Piehl F., Nelson D.W., Bellander B.-M. (2015). Comparative Assessment of the Prognostic Value of Biomarkers in Traumatic Brain Injury Reveals an Independent Role for Serum Levels of Neurofilament Light. PLoS ONE.

[107] Gao W., Zhang Z., Lv X., Wu Q., Yan J., Mao G., Xing W. (2020). Neurofilament Light Chain Level in Traumatic Brain Injury. Medicine.

[108] Kuhle J., Barro C., Andreasson U., Derfuss T., Lindberg R., Sandelius Å., Liman V., Norgren N., Blennow K., Zetterberg H. (2016). Comparison of Three Analytical Platforms for Quantification of the Neurofilament Light Chain in Blood Samples: ELISA, Electrochemiluminescence Immunoassay and Simoa. Clin. Chem. Lab. Med..

[109] Alhajj M., Zubair M., Farhana A. (2023). Enzyme Linked Immunosorbent Assay. StatPearls.

[110] Mondello S., Muller U., Jeromin A., Streeter J., Hayes R.L., Wang K.K. (2011). Blood-Based Diagnostics of Traumatic Brain Injuries. Expert Rev. Mol. Diagn..

[111] He X.-Y., Dan Q.-Q., Wang F., Li Y.-K., Fu S.-J., Zhao N., Wang T.-H. (2018). Protein Network Analysis of the Serum and Their Functional Implication in Patients Subjected to Traumatic Brain Injury. Front. Neurosci..

[112] Hubbard W.B., Banerjee M., Vekaria H., Prakhya K.S., Joshi S., Wang Q.J., Saatman K.E., Whiteheart S.W., Sullivan P.G. (2021). Differential Leukocyte and Platelet Profiles in Distinct Models of Traumatic Brain Injury. Cells.

[113] Gorsky A., Monsour M., Nguyen H., Castelli V., Lee J.-Y., Borlongan C.V. (2023). Metabolic Switching of Cultured Mesenchymal Stem Cells Creates Super Mitochondria in Rescuing Ischemic Neurons. Neuromolecular Med..

[114] Underwood E., Redell J.B., Zhao J., Moore A.N., Dash P.K. (2020). A Method for Assessing Tissue Respiration in Anatomically Defined Brain Regions. Sci. Rep..

[115] Hubbard W.B., Spry M.L., Gooch J.L., Cloud A.L., Vekaria H.J., Burden S., Powell D.K., Berkowitz B.A., Geldenhuys W.J., Harris N.G. (2021). Clinically Relevant Mitochondrial-Targeted Therapy Improves Chronic Outcomes after Traumatic Brain Injury. Brain.

[116] Lin C., Li N., Chang H., Shen Y., Li Z., Wei W., Chen H., Lu H., Ji J., Liu N. (2020). Dual Effects of Thyroid Hormone on Neurons and Neurogenesis in Traumatic Brain Injury. Cell Death Dis..

[117] Schmidt C.A., Fisher-Wellman K.H., Neufer P.D. (2021). From OCR and ECAR to Energy: Perspectives on the Design and Interpretation of Bioenergetics Studies. J. Biol. Chem..

[118] Nolfi-Donegan D., Braganza A., Shiva S. (2020). Mitochondrial Electron Transport Chain: Oxidative Phosphorylation, Oxidant Production, and Methods of Measurement. Redox Biol..

[119] Desousa B.R., Kim K.K., Jones A.E., Ball A.B., Hsieh W.Y., Swain P., Morrow D.H., Brownstein A.J., Ferrick D.A., Shirihai O.S. (2023). Calculation of ATP Production Rates Using the Seahorse XF Analyzer. EMBO Rep..

[120] Mookerjee S.A., Nicholls D.G., Brand M.D. (2016). Determining Maximum Glycolytic Capacity Using Extracellular Flux Measurements. PLoS ONE.

[121] Kramer P.A., Ravi S., Chacko B., Johnson M.S., Darley-Usmar V.M. (2014). A Review of the Mitochondrial and Glycolytic Metabolism in Human Platelets and Leukocytes: Implications for Their Use as Bioenergetic Biomarkers. Redox Biol..

[122] Chacko B.K., Smith M.R., Johnson M.S., Benavides G., Culp M.L., Pilli J., Shiva S., Uppal K., Go Y.-M., Jones D.P. (2019). Mitochondria in Precision Medicine; Linking Bioenergetics and Metabolomics in Platelets. Redox Biol..

[123] Shandley S., Wolf E.G., Schubert-Kappan C.M., Baugh L.M., Richards M.F., Prye J., Arizpe H.M., Kalns J. (2017). Increased Circulating Stem Cells and Better Cognitive Performance in Traumatic Brain Injury Subjects Following Hyperbaric Oxygen Therapy. Undersea Hyperb. Med..

[124] Borlongan C. (2011). Bone Marrow Stem Cell Mobilization in Stroke: A ‘Bonehead’ May Be Good after All!. Leukemia.

[125] Kucia M., Reca R., Campbell F.R., Zuba-Surma E., Majka M., Ratajczak J., Ratajczak M.Z. (2006). A Population of Very Small Embryonic-like (VSEL) CXCR4+SSEA-1+Oct-4+ Stem Cells Identified in Adult Bone Marrow. Leukemia.

[126] Gravina A., Tediashvili G., Rajalingam R., Quandt Z., Deisenroth C., Schrepfer S., Deuse T. (2023). Protection of Cell Therapeutics from Antibody-Mediated Killing by CD64 Overexpression. Nat. Biotechnol..

[127] Dawson N.A.J., Lamarche C., Hoeppli R.E., Bergqvist P., Fung V.C.W., McIver E., Huang Q., Gillies J., Speck M., Orban P.C. (2019). Systematic Testing and Specificity Mapping of Alloantigen-Specific Chimeric Antigen Receptors in Regulatory T Cells. JCI Insight.

[128] Krueger W.H., Tanasijevic B., Norris C., Tian X.C., Rasmussen T.P. (2013). Oct4 Promoter Activity in Stem Cells Obtained through Somatic Reprogramming. Cell. Reprogramming.

[129] Smith R.C.G., Stumpf P.S., Ridden S.J., Sim A., Filippi S., Harrington H.A., MacArthur B.D. (2017). Nanog Fluctuations in Embryonic Stem Cells Highlight the Problem of Measurement in Cell Biology. Biophys. J..

[130] Gang E.J., Bosnakovski D., Figueiredo C.A., Visser J.W., Perlingeiro R.C.R. (2006). SSEA-4 Identifies Mesenchymal Stem Cells from Bone Marrow. Blood.

[131] Picot J., Guerin C.L., Le Van Kim C., Boulanger C.M. (2012). Flow Cytometry: Retrospective, Fundamentals and Recent Instrumentation. Cytotechnology.

[132] Chen Y., Wang L., You W., Huang F., Jiang Y., Sun L., Wang S., Liu S. (2022). Hyperbaric oxygen therapy promotes consciousness, cognitive function, and prognosis recovery in patients following traumatic brain injury through various pathways. Front. Neurol..

[133] Barman A., Chatterjee A., Bhide R. (2016). Cognitive Impairment and Rehabilitation Strategies After Traumatic Brain Injury. Indian. J. Psychol. Med..

[134] Liu Y.S., Liu Z.B., Yang Z., Zhao L., Li H.L. (2022). Clinical efficacy of hyperbaric oxygen combined with different timings of right median-nerve electrical stimulation in patients with brain injury-induced disorders of consciousness. Brain Behav..

[135] Boussi-Gross R., Golan H., Fishlev G., Bechor Y., Volkov O., Bergan J., Friedman M., Hoofien D., Shlamkovitch N., Ben-Jacob E. (2013). Hyperbaric oxygen therapy can improve post concussion syndrome years after mild traumatic brain injury—Randomized prospective trial. PLoS ONE.

[136] Hadanny A., Abbott S., Suzin G., Bechor Y., Efrati S. (2018). Effect of hyperbaric oxygen therapy on chronic neurocognitive deficits of post-traumatic brain injury patients: Retrospective analysis. BMJ Open.

[137] Heyburn L., Abutarboush R., Goodrich S., Urioste R., Batuure A., Statz J., Wilder D., Ahlers S.T., Long J.B., Sajja V. (2019). Repeated Low-Level Blast Overpressure Leads to Endovascular Disruption and Alterations in TDP-43 and Piezo2 in a Rat Model of Blast TBI. Front. Neurol..

[138] Gama Sosa M.A., De Gasperi R., Perez Garcia G.S., Perez G.M., Searcy C., Vargas D., Spencer A., Janssen P.L., Tschiffely A.E., McCarron R.M. (2019). Low-level blast exposure disrupts gliovascular and neurovascular connections and induces a chronic vascular pathology in rat brain. Acta Neuropathol. Commun..

[139] Marcinkowska A.B., Mankowska N.D., Kot J., Winklewski P.J. (2022). Impact of Hyperbaric Oxygen Therapy on Cognitive Functions: A Systematic Review. Neuropsychol. Rev..

[140] Parr N.J., Anderson J., Veazie S. (2021). Evidence Brief: Hyperbaric Oxygen Therapy for Traumatic Brain Injury and/or Post-traumatic Stress Disorder. VA Evidence-Based Synthesis Program Reports.

[141] Heyboer M., Sharma D., Santiago W., McCulloch N. (2017). Hyperbaric Oxygen Therapy: Side Effects Defined and Quantified. Adv. Wound Care.

